# Metal Oxide Nanoparticles Induce Unique Inflammatory Footprints in the Lung: Important Implications for Nanoparticle Testing

**DOI:** 10.1289/ehp.1002201

**Published:** 2010-08-20

**Authors:** Wan-Seob Cho, Rodger Duffin, Craig A. Poland, Sarah E.M. Howie, William MacNee, Mark Bradley, Ian L. Megson, Ken Donaldson

**Affiliations:** 1 ELEGI (The Edinburgh Lung and the Environment Group Initiative), Centre for Inflammation Research, University of Edinburgh, Edinburgh, United Kingdom;; 2 Immunology Group, Centre for Inflammation Research and; 3 School of Chemistry, University of Edinburgh, Edinburgh, United Kingdom;; 4 Free Radical Research Facility, Department of Diabetes and Cardiovascular Science, Centre for Health Science, University of the Highlands and Islands, Inverness, United Kingdom

**Keywords:** eosinophilic inflammation, intratracheal instillation, *in vitro* assay, *in vivo* assay, lymphocytic inflammation, metal oxide nanoparticles, neutrophilic inflammation, risk assessment, surface area dose, Wistar rat

## Abstract

**Background:**

Metal oxide nanoparticles (NPs) have been widely used in industry, cosmetics, and biomedicine.

**Objectives:**

We examined hazards of several well-characterized high production volume NPs because of increasing concern about occupational exposure via inhalation.

**Methods:**

A panel of well-characterized NPs [cerium oxide (CeO_2_NP), titanium dioxide (TiO_2_NP), carbon black (CBNP), silicon dioxide (SiO_2_NP), nickel oxide (NiONP), zinc oxide (ZnONP), copper oxide (CuONP), and amine-modified polystyrene beads] was instilled into lungs of rats. We evaluated the inflammation potencies of these NPs 24 hr and 4 weeks postinstillation. For NPs that caused significant inflammation at 24 hr, we then investigated the characteristics of the inflammation. All exposures were carried out at equal-surface-area doses.

**Results:**

Only CeO_2_NP, NiONP, ZnONP, and CuONP were inflammogenic to the lungs of rats at the high doses used. Strikingly, each of these induced a unique inflammatory footprint both acutely (24 hr) and chronically (4 weeks). Acutely, patterns of neutrophil and eosinophil infiltrates differed after CeO_2_NP, NiONP, ZnONP, and CuONP treatment. Chronic inflammatory responses also differed after 4 weeks, with neutrophilic, neutrophilic/lymphocytic, eosinophilic/fibrotic/granulomatous, and fibrotic/granulomatous inflammation being caused respectively by CeO_2_NP, NiONP, ZnONP, and CuONP.

**Conclusion:**

Different types of inflammation imply different hazards in terms of pathology, risks, and risk severity. *In vitro* testing could not have differentiated these complex hazard outcomes, and this has important implications for the global strategy for NP hazard assessment. Our results demonstrate that NPs cannot be viewed as a single hazard entity and that risk assessment should be performed separately and with caution for different NPs.

Compared with bulk materials, nanoparticles (NPs) have unique and novel properties and thus offer great opportunities for development of new industrial applications (Borm et al. 2006). Many NPs are already in use or have potential to be widely used in a range of applications (Nohynek et al. 2007). Maynard et al. (2006) and Nel et al. (2006) have called for risk assessment for the environment and humans before widespread industrial application of NPs. Such risk assessment requires hazard identification and dose–response data.

*In vitro* assays have limitations and have not necessarily been validated for NPs (Kroll et al. 2009). In addition, the traditional mass dose does not well reflect the biologically effective dose for NPs, and surface area combined with surface reactivity is more likely a better descriptor of potential to cause inflammation at the site of particle deposition (Donaldson et al. 2008; Duffin et al. 2007; Tran et al. 2000).

The commonly proposed pathogenic mechanisms initiated by NPs are dominated by inflammation-driven effects, including fibrosis, oxidative stress, and DNA damage, making inflammation a target for toxicological testing (Borm et al. 2006; Lu et al. 2009; Nel et al. 2006, 2009). Inflammation is a complex, concerted group of responses that, although defensive against infection, is harmful when induced chronically by environmental stimuli such as inhaled particles (Donaldson et al. 2006; Mroz et al. 2008; Oberdorster et al. 2005). The type, harmfulness, and outcome of inflammation vary depending on the nature of the stimulus initiating the inflammation; the affected tissue; the nature of the cellular exudates; its chronicity, severity, and potential to resolve; and the genetic susceptibility of the individual.

Inflammation cannot be replicated by *in vitro* models because it depends on an intact vascular system and a huge assortment of cellular and humoral interactions. Although *in vitro* studies with NPs can claim to demonstrate “proinflammatory effects,” such studies cannot demonstrate anything more than a general indication that such articles are likely to elicit “some sort” of inflammation; they cannot predict the form of inflammation or its tempo, persistence, or tendency to resolve.

We studied the acute pulmonary toxicity of a large panel of NPs [cerium oxide (CeO_2_NP), titanium dioxide (TiO_2_NP), carbon black (CBNP), silicon dioxide (SiO_2_NP), nickel oxide (NiONP), zinc oxide (ZnONP), copper oxide (CuONP), and amine-modified polystyrene beads (Beads)] and found that four of them possessed significant acute inflammogenicity: CeO_2_NP, NiONP, ZnONP, and CuONP. We went on to study these four NPs further and found very different patterns of inflammation (in terms of time), resolution, and cellular exudates with each of the four different particle types.

## Materials and Methods

### Characterization of NPs

We used a panel of eight NPs (CeO_2_NP, TiO_2_NP, CBNP, SiO_2_NP, NiONP, ZnONP, CuONP, and Beads) purchased from commercial sources, as noted in Table 1. These NPs are typical of those most commonly used in industry. The surface area of NPs [Brunauer-Emmett-Teller (BET) method] was determined by ParticlesCIC Ltd. (Leeds, UK) using a Micromeritics TriStar 3000 analyzer (Micromeritics Ltd., Dunstable, Bedfordshire, UK). All exposures took place using NPs dispersed in rat serum. NP stock solution was prepared at 6,000 cm^2^/mL in distilled water and sonicated with a probe sonicator (Philip Harris Scientific, Lichfield, UK) to break up aggregates. Rat serum (final concentration, 5%) was added for dispersion. The stock solution of NPs was then diluted with phosphate-buffered saline (PBS) to the final concentration. The hydrodynamic size and zeta potential of the NPs were analyzed in PBS containing 5% rat serum with a Brookhaven 90Plus particle size analyzer (Brookhaven Instruments Corp., Holtsville, NY, USA) and a Zetasizer-Nano ZS instrument (Malvern Instruments Ltd., Malvern Hills, UK), respectively. The levels of endotoxin in the NP suspension were evaluated by the *Limulus* amebocyte lysate assay (Cambrex Corp., Walkersville, MD, USA).

### Intratracheal instillation of NPs and bronchoalveolar lavage

Female Wistar rats (200–250 g) obtained from Harlan Laboratories (Hillcrest, UK) were maintained and handled under a specific license granted by the UK Home Office to one of the authors (K.D.) that ensures humane treatment and alleviation of suffering in all animal experiments. NPs dispersed in saline containing 5% rat serum were prepared at surface area doses of 100 and 300 cm^2^/mL. The hydrodynamic size of the particles, as instilled (Table 1), confirms that these were not singlet NPs but aggregates whose size was well within the respirable size range (which is a requirement for studies on any inhalation hazard). For controls, we used nontreated (sham) rats and rats instilled with saline containing 5% rat serum (vehicle) (*n* = 5 animals per group). Intratracheal instillation of NPs and lavages were performed using methods previously described by Lu et al. (2009). For 24-hr studies, we instilled eight NPs at doses of 50 and 150 cm^2^/rat. In addition, CeO_2_NP, NiONP, ZnONP, and CuONP, which were inflammogenic at 24 hr, were used for chronic studies; chronic toxicity study was also performed at the same doses.

### Effects of different dispersant on inflammogenicity by ZnONP

To evaluate the serum effects on the inflammogenicity of ZnONP, the NPs were dispersed with rat serum, human serum, or bronchoalveolar lavage fluid (BALF). Then, the ZnONPs were instilled into rats at 150 cm^2^/rat for 24 hr and a differential cell count was performed on the BALF.

### Preparation of BALF

BALF samples were prepared as described previously (Lu et al. 2009). Briefly, 10,000 cells were centrifuged onto glass slides; cells were then fixed with methanol and stained using Diff-Quik. We measured lactate dehydrogenase (LDH) levels in BALF using the Cytotoxicity Detection Kit (Roche Diagnostics Ltd., Burgess Hill, UK) according to the manufacturer’s instructions. Data are expressed as fold change compared with the vehicle control. Concentration of total protein in BALF was measured using a bicinchoninic acid (BCA) assay (Sigma-Aldrich, Gillingham, Dorset, UK) according to the manufacturer’s instructions. Tumor necrosis factor-α (TNF-α), interleukin (IL)-1β, macrophage inflammatory protein-2 (MIP-2), eotaxin, and interferon-γ (IFN-γ), all from the Quantikine Kit (R&D Systems Europe Ltd., Abingdon, UK), and IL-13 from Invitrogen (Camarillo, CA, USA) were measured in nondiluted BALF following the manufacturers’ directions.

### Histology and immunohistochemistry

For histological analysis, lungs fixed with 10% neutral buffered formalin were embedded in paraffin, sectioned, and stained with hematoxylin and eosin (H&E). We performed picrosirius red (PSR) staining for analysis of fibrosis. Briefly, sections were deparaffinized and hydrated with distilled water for 10 min. Sections were stained with 0.1% PSR for 90 min and then dehydrated and mounted. Immunohistochemical staining for CD3 as a T-cell marker and CD45RA as a B-cell marker was performed in the lungs of NiONP at 4 weeks postinstillation on paraffin sections. Endogenous peroxidase activity was quenched with 3% hydrogen peroxide at room temperature for 15 min. For antigen retrieval, Borg Decloaker (Biocare Medical Inc., Walnut Creek, CA, USA) was applied for 2 min. Slides were then blocked with normal goat serum, and anti-rat CD3 antibody or anti-rat CD45RA antibody (both from AbD Serotec, Oxford, UK) was applied at 1:100 dilution. Slides were washed three times and incubated for 30 min at room temperature with anti-mouse IgG from the Envision kit (Dako, Cambridgeshire, UK). After slides were washed three times, diaminobenzidine substrate (Vector Laboratories, Peterborough, UK) was applied.

### Vector diagram

We used vector diagrams to show the different patterns of inflammation. Parameters were grouped by neutrophilic [polymorphonuclear leukocytes (PMN), MIP-2, and IL-1β], cytotoxic (LDH and total protein), eosinophilic (eosinophils, eotaxin, and IL-13), and lymphocytic (lymphocytes and IFN-γ) inflammation. A template for drawing vector diagrams was transferred from Microsoft Office Excel (version 2007; Microsoft UK, Reading, UK) to Adobe Photoshop (version CS3; Adobe Consulting, London, UK). All data were inserted into the diagrams in the units originally derived, as given in the figures and tables. Each vector was then drawn and colored using Adobe Photoshop.

### Statistical analysis

Data are expressed as mean ± SD (*n* = 4) and were analyzed with GraphPad InStat software (version 3; GraphPad Software, Inc., La Jolla, CA, USA). We used one-way analysis of variance with post hoc Tukey’s pairwise comparisons to compare each treatment group. The value of *p* < 0.05 was taken to be statistically significant.

## Results

### Characterization of NPs

The physicochemical characteristics of the panel of eight NPs are summarized in Table 1. Hydrodynamic size showed that all NPs had small aggregates, but the hydrodynamic size of SiO_2_NP and ZnONP (~ 300 nm) was larger than that of the other NPs (~ 100 nm). All NPs except Beads were negatively charged.

### Cytological analysis of the BALF

#### Twenty-four hours

**Figure 1:**
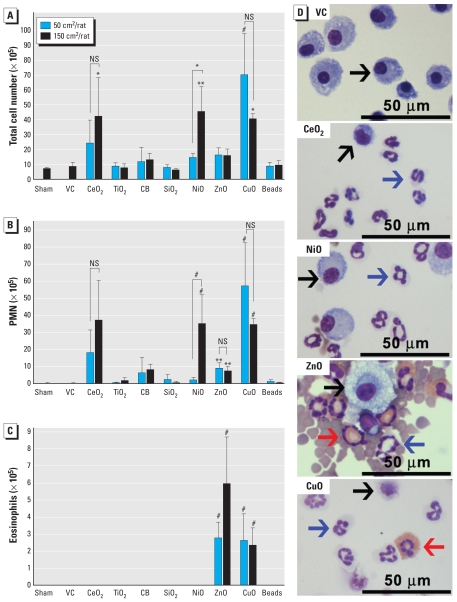
Differential cell counts and images of BALF cells from rats 24 hr after intratracheal instillation of NPs (50 or 150 cm^2^/rat). Number of (*A*) total cells, (*B*) PMN, and (*C*) eosinophils. (*D*) Photomicrographs of BALF cells (cytospin preparations stained with Diff-Quik) from rats treated with 150 cm^2^ NPs. Black arrows indicate macrophages; blue arrows, PMN; red arrows, eosinophils. Abbreviations: NS, not significant; VC, vehicle control. For *A–C*, data are mean ± SD (*n* = 4 per group). Each treatment group was compared with vehicle control for statistical significance, whereas high-dose groups were compared with low-dose group to evaluate dose dependency. *p < 0.05, **p < 0.01, and ^#^*p* < 0.001.

Total cells in BALF of rats exposed to CeO_2_NP, NiONP, or CuONP were significantly increased (Figure 1) compared with vehicle controls. Total cells were also significantly higher in rats treated with 150-cm^2^ versus 50-cm^2^ NiONP. The numbers of PMN in BALF were significantly higher than those in vehicle controls after exposure to CeO_2_NP, NiONP, ZnONP, or CuONP, but we saw a significant positive dose response only with NiONP. Eosinophils were significantly increased in BALF of rats treated with ZnONP or CuONP. Other NPs did not increase the number of eosinophils in BALF.

#### Four weeks

**Figure 2:**
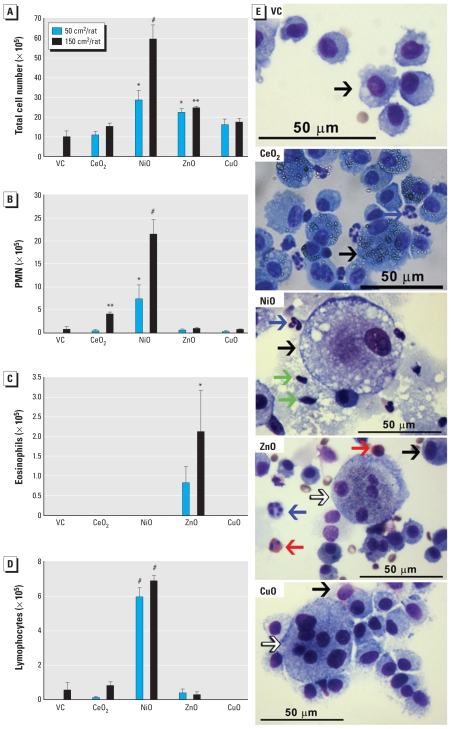
Differential cell counts and images of BALF cells from rats 4 weeks after intratracheal instillation of NPs (50 or 150 cm^2^/rat). Number of (*A*) total cells, (*B*) PMN, (*C*) eosinophils, and (*D*) lymphocytes. (*E*) Photomicrographs of BALF cells (cytospin preparations stained with Diff-Quik) from rats treated with 150 cm^2^ NP. Black arrows indicate macrophages; blue arrows, PMN; red arrows, eosinophils; green arrows, lymphocytes; white arrows, giant cells. Abbreviations: NS, not significant; VC, vehicle control. For *A–D*, data are mean ± SD (*n* = 4 per group). *p < 0.05, **p < 0.01, and ^#^*p* < 0.001, compared with the vehicle control.

Total cell numbers were significantly higher after NiONP or ZnONP treatment compared with vehicle controls (Figure 2). PMN were significantly increased with CeO_2_NP or NiONP treatment, and lymphocytes were significantly increased in the NiONP treatment groups. In addition, eosinophils were still significantly present in BALF at 4 weeks in the high-dose ZnONP group but not in the low-dose group. We observed giant cells, a marker for chronic granulomatous inflammation, in lungs after treatment with ZnONP or CuONP. Interestingly, the neutrophilic inflammation caused by NiONP was much more severe at 4 weeks than at 24 hr. Low-dose NiONP recruited the same types of inflammatory cells in the BALF at 4 weeks after instillation as the high dose. We observed no significant increases in inflammatory cells 4 weeks after treatment with CuONP.

### LDH and total protein concentration in BALF

#### Twenty-four hours

LDH, a marker of cell death, and total protein, a marker of cell permeability, were significantly increased in BALF relative to vehicle controls 24 hr after treatment with CeO_2_NP, NiONP, ZnONP, or CuONP [see Supplemental Material, Figure 1 (doi:10.1289/ehp.1002201)]. These findings were paradoxical for ZnONP given that treatment was not associated with a very substantial leukocyte influx. We observed significant positive dose responses for LDH and total protein levels after NiONP and ZnONP treatment. Protein concentration was significantly higher and LDH concentration was significantly lower for high-dose CuONP than for low-dose CuONP. Total protein and LDH levels were comparable between high- and low-dose CeO_2_NP groups.

#### Four weeks

LDH was significantly increased over controls 4 weeks after treatment in the high-dose CeO_2_NP group and both NiONP groups [see Supplemental Material, Figure 2 (doi:10.1289/ehp.1002201)]. Total protein levels also were significantly increased in both NiONP treatment groups. In contrast, low-dose NiONP was not associated with significant increases in LDH or total protein levels 24 hr after instillation.

### Histological analysis

#### Twenty-four hours

**Figure 3:**
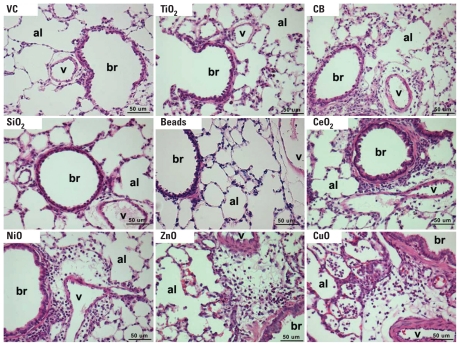
Photomicrographs showing lung histology 24 hr after intratracheal instillation of NPs (150 cm^2^/rat). Abbreviations: al, alveolus; br, bronchiole; v, blood vessel; VC, vehicle control. Instillation of CeO_2_NP, NiONP, ZnONP, and CuONP induced multifocal inflammatory cell infiltration in the alveoli, peribronchiolar, and perivascular regions. Tissues were stained with H&E; each panel is representative of lung lesions observed in the same treatment group in this study. Bars = 50 μm.

Consistent with the BALF cytology results, CeO_2_NP, NiONP, ZnONP, and CuONP induced inflammation in lung tissues (Figure 3). CeO_2_NP and NiONP treatments were associated with mild to moderate neutrophilic infiltration in alveoli and in peribronchial and perivascular regions. ZnONP treatment was associated with mild neutrophilic and severe eosinophilic inflammation in alveoli and peribronchial and perivascular regions, with moderate hemorrhage. CuONP treatment was associated with severe neutrophilic and mild eosinophilic inflammation in alveoli and in peribronchial and perivascular regions, with mild hemorrhage and moderate type II cell hyperplasia. TiO_2_NP, SiO_2_NP, and Beads produced no detectable inflammation in the lung, whereas CBNP treatment was associated with mild perivascular neutrophilic inflammation.

#### Four weeks

**Figure 4:**
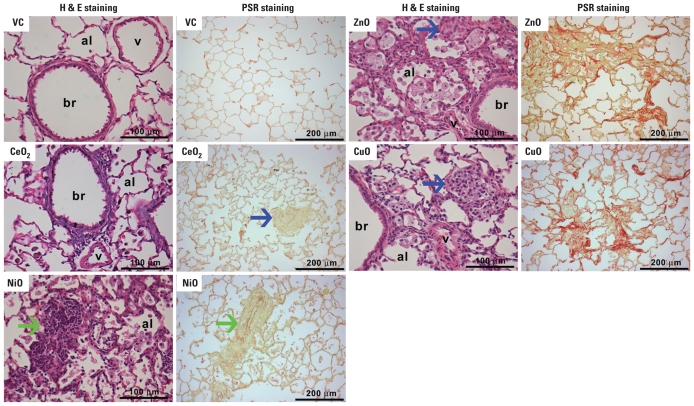
Photomicrographs of H&E (histology) and PSR (fibrosis) staining of lungs from rats 4 weeks after treatment with NPs (150 cm^2^/rat). Abbreviations: al, alveolus; br, bronchiole; v, blood vessel; VC, vehicle control. Each panel is representative of lung lesions observed in the same treatment group in this study. Extremely rare small granulomas were observed in the CeO_2_NP–exposed lungs; see the example in Supplemental Material, Figure 4 (doi:10.1289/ehp.1002201). PSR staining showed that ZnONP and CuONP induced severe collagen deposition, whereas CeO_2_NP and NiONP showed no response. Blue arrows indicate granulomatous inflammation; green arrows, lymphocyte aggregation.

Histological analysis of lung sections obtained 4 weeks after NiONP installation showed lipoproteinaceous material in the alveolar spaces, foamy macrophages (presumed to contain lipoprotein) in the alveolar spaces (Figure 4), and many organized lymphocyte aggregates in the perivascular and peribronchial interstitium that consisted of B cells and T cells [see Supplemental Material, Figure 3 (doi:10.1289/ehp.1002201)]. Interestingly, we did not observe giant cells and fibrosis—which are commonly associated with chronic inflammation—after NiONP treatment. CeO_2_NP induced minimal to mild neutrophilic inflammation and rare granulomas in alveoli and interstitium (Figure 4; see also Supplemental Material, Figure 4). In contrast, ZnONP induced alveolar and interstitial inflammatory cell infiltration, including eosinophils, macrophages, and giant cells, with severe fibrotic lesions that were predominantly in collapsed and contracted alveoli. CuONP induced granulomatous inflammation in alveoli and also induced interstitial fibrosis that was less severe than that associated with ZnONP.

### Measurement of proinflammatory mediators

To evaluate the cytokine profile underlying inflammation, we selected and measured representative inflammatory mediators for neutrophilic (TNF-α, IL-1β, and MIP-2), eosinophilic (eotaxin and IL-13), and lymphocytic (IFN-γ) inflammation in BALF.

#### Twenty-four hours

We observed no significant difference in TNF-α or IFN-γ in any treatment group compared with the vehicle control (data not shown). IL-1β was significantly increased in the high-dose CeO_2_NP, NiONP, and ZnONP and high- and low-dose CuONP treatment groups [Table 2; see also Supplemental Material, Figure 5 (doi:10.1289/ehp.1002201)]. MIP-2 levels in BALF were significantly increased in the low- and high-dose CeO_2_NP, NiONP, and CuONP groups, but MIP-2 was not increased with either dose of ZnONP. ZnONP treatment was associated with significant increases in eotaxin and IL-13, consistent with the severe acute eosinophilia observed after ZnONP treatment. CuONP treatment, which resulted in modest eosinophilia, was associated with significantly increased eotaxin only.

#### Four weeks

At 4 weeks after instillation, levels of MIP-2 were significantly increased in the high-dose CeO_2_NP group and both NiONP dose groups [see Supplemental Material, Figure 6 (doi:10.1289/ehp.1002201)]. Levels of IFN-γ were significantly increased after treatment with low- and high-dose NiONP and high-dose ZnONP. In contrast to the increases in IL-1β and MIP-2 observed after 24-hr treatment with CuONP, the levels of these cytokines 4 weeks after installation were not significantly different from vehicle controls.

### Effects of dispersion media on eosinophil recruitment by ZnONP

To evaluate the effects of rat serum on the recruitment of eosinophils, we instilled ZnONP with several dispersants. After 24 hr, ZnONP treatment was associated with comparable numbers of eosinophils in BALF when dispersed using rat serum (5.95 ± 2.74, × 10^5^), human serum (5.52 ± 1.45, × 10^5^), and rat BALF (5.40 ± 1.57, × 10^5^).

## Discussion

We dispersed NPs in 5% rat serum using an equal-surface-area dose as the exposure metric and instilled mass doses ranging from 50 to 1,250 μg, which are roughly comparable with the predicted alveolar retained mass of 50 μg that would result from 1 month of exposure to a 250-μg/m^3^ cloud of NPs with aerodynamic diameter of 0.25 μm (consistent with typical NPs included in our study) according to the multiple-path particle dosimetry deposition model (Cassee et al. 2002). No single characteristic, including zeta potential, clearly predicted the inflammogenic potency of the eight NPs.

**Figure 5:**
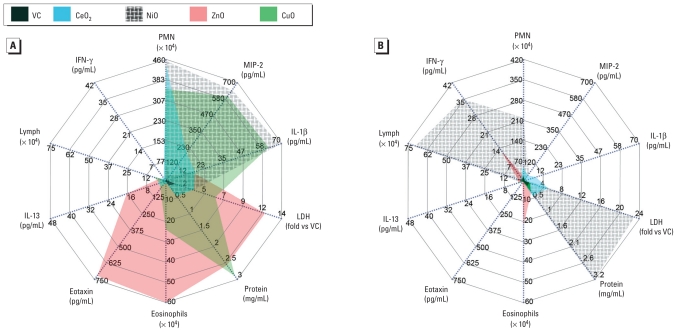
Vector diagrams showing trends and types of acute (*A*) and chronic (*B*) inflammation after instillation of NPs (150 cm^2^/rat). The various inflammatory parameters are shown as vectors, where radial axes depict the relative magnitude of any vector quantity. By joining the vector quantities, a unique pattern is produced for any treatment. The left upper quadrant shows lymphocytic parameters (lymphocytes and IFN-γ); right upper quadrant, neutrophilic parameters (PMN, MIP-2, and IL-1β); lower right quadrant, cytotoxic parameters (LDH and total protein); and lower left quadrant, eosinophilic parameters (eosinophils, eotaxin, and IL-13). VC, vehicle control. In *A* (24 hr after instillation), CeO_2_NP and NiONP showed neutrophilic inflammation with mild cytotoxicity. ZnONP and CuONP, respectively, showed eosinophilic inflammation with severe cytotoxicity and neutrophilic/eosinophilic inflammation with severe cytotoxicity to the lungs of rats. In *B*) (4 weeks after instillation), CeO_2_NP still showed neutrophilic/mild cytotoxic inflammation. In contrast, NiONP showed neutrophilic/lymphocytic inflammation with severe cytotoxicity, and ZnONP and CuONP showed eosinophilic inflammation without cytotoxicity and no inflammatory responses, respectively.

Using the rat model, we found that four metal oxide NPs each induced a different type of inflammation characterized by different types of infiltrating cells, inflammatory mediators, time course, and cytotoxicity. The vector diagrams in Figure 5 show the “inflammatory footprints” of each NP 24 hr and 4 weeks after instillation. After 24 hr of exposure, CeO_2_NP and NiONP were associated with a neutrophilic/mild cytotoxic pattern; ZnONP was associated with an eosinophilic/severe cytotoxic pattern; and CuONP was associated with a neutrophilic/eosinophilic/severe cytotoxic pattern. Four weeks after instillation, differences among NPs and from the 24-hr patterns were evident from the chronic inflammation patterns we observed, including a modest residual neutrophilic/mild cytotoxic inflammation with CeO_2_NP; a greatly amplified immunological/severely cytotoxic inflammation with NiONP; and the almost total resolution of acute inflammation with CuONP. We also observed a modest residual eosinophilic/immunological signal in BALF from ZnONP-exposed rats after 4 weeks.

It is important to note the contrast between the BALF results and the histology of the lung sections. Although the BALF inflammatory profile had waned dramatically by 4 weeks in both ZnONP- and CuONP-exposed rats, the histological assessments showed fibrosis in both cases, although the specific histological pattern differed between the ZnONP and CuONP groups. In contrast, the inflammation in the NiONP group, which had amplified greatly between 24 hr and 4 weeks, was not associated with fibrosis, although alveolar lipoproteinosis was present indicating severe, ongoing epithelial injury. It is possible, even likely, that fibrosis could develop in the longer term in NiONP-exposed lungs, as documented in previous studies with NiO particles and NPs showing late-developing pulmonary fibrosis (Ogami et al. 2009; Ozaki et al. 2002). The histological lesions 24 hr and 4 weeks after CeO_2_NP instillation were consistent with BALF analysis (neutrophilic and mild cytotoxic inflammation) except for the rare granulomas present at 4 weeks, which seem to be associated with areas of high particle deposition. We used instillation in this study as a substitute for inhalation; although instillation is known to produce a localized high dose and a high dose rate, it is a useful method for comparing responses to different particle types (Driscoll et al. 2000).

CeO_2_NP- and NiONP-induced neutrophilic/mildly cytotoxic inflammation at 24 hr was associated with elevated IL-1β, MIP-2, and LDH in BALF, and both IL-1β and MIP-2 are mediators of acute neutrophilic inflammation (Ulich et al. 1991; Wolpe et al. 1989). Four weeks after CeO_2_NP instillation, a mild neutrophilic/cytotoxic inflammation associated with modestly increased MIP-2 expression was still evident. CeO_2_NP has been used as a fuel additive for diesel engines (B. Park et al. 2008), and *in vitro* studies have shown that CeO_2_NP increased production of reactive oxygen species in A549 cells (Lin et al. 2006) and BEAS-2B cells (E.J. Park et al. 2008). In contrast, CeO_2_NP has been reported to be noninflammatory (Lu et al. 2009) after pulmonary instillation, or cytoprotective *in vitro* by means of scavenging free radicals or reactive oxygen species (Hirst et al. 2009). The discrepancies between our findings in this study and those in previous studies might be due to improved dispersion in the present study, which greatly increases the surface area compared with aggregated NPs (Monteiller et al. 2007).

NiONP induced chronic neutrophilic/lymphocytic/severely cytotoxic inflammation 4 weeks after instillation accompanied by increased MIP-2, IFN-γ, and LDH in BALF (Table 2). At this time point, the alveolar lipoproteinosis evident in NiONP-exposed lungs was reflected in very high protein and LDH levels in the BALF. IFN-γ is linked with lymphocytic inflammation (Bradley et al. 1996). Consistent with our findings, increased levels of neutrophils and macrophages have been observed from 3 days to 3 months after instillation of agglomerated NiONP suspended in distilled water in a previous study using Wistar rats (Nishi et al. 2009; Ogami et al. 2009). However, Nishi et al. (2009) did not conclude that worsening inflammation was driven by a developing allergic response, as suggested by our findings. The difference in the two studies might be due to the dispersion of NPs because, in our previous study (Lu et al. 2009), we reported that intratracheal instillation of NiONP without dispersion into Wistar rats induced acute neutrophilic inflammation in the lungs.

The eosinophilic/severely cytotoxic inflammation seen with ZnONP in BALF at 24 hr was associated with very high levels of eotaxin, IL-13, and LDH (Table 2). Eotaxin and IL-13 are chemokines principally recruiting eosinophils, a component of the inflammatory exudates in asthmatic lung diseases (Conroy and Williams 2001; Wills-Karp 2004). The granulomatous/eosinophilic inflammation seen in ZnONP-exposed lungs at 4 weeks was associated with increased IFN-γ levels. Until now, a limited number of *in vivo* studies have been performed on the pulmonary toxicity of ZnONP. Intratracheal instillation of both nanometer-size ZnO (50–70 nm) and micrometer-size ZnO (< 1,000 nm) in rats resulted in severe cytotoxicity and neutrophilic (but not eosinophilic) inflammation that peaked at 24 hr and resolved by 4 weeks after instillation (Sayes et al. 2007; Warheit et al. 2009). In our previous study (Lu et al. 2009), we instilled 90–210 nm ZnO particles and found PMN, but no eosinophils, in BALF at 24 hr. In the present study we focused on the cellular nature, types, and mechanism of inflammation with nanometer-size ZnO (< 10 nm). It is notable that, in a human subject, accidental exposure to ZnO induced metal fume fever that recruited high levels of eosinophils into BALF (Castet and Bouillard 1992).

In the present study, exposure to CuONP was associated with neutrophilic/eosinophilic/severely cytotoxic inflammation and increased levels of IL-1β, MIP-2, eotaxin, and LDH in BALF 24 hr after instillation. The granulomatous inflammation seen 4 weeks after CuONP exposure was not associated with any increase in the measured cytokines or LDH. In a previous study (Yokohira et al. 2008), CuONP was also very cytotoxic and induced severe neutrophilic and granulomatous inflammation when instilled into lungs of rats.

The finding of eosinophils in the cellular exudates of rats exposed to two of the NPs used here was unexpected. The rats used in our study were from a reputable supplier (Harlan Laboratories), and we have not encountered eosinophils in the lavage of rats instilled with numerous other dusts (e.g., asbestos, coal mine dust, glass fibers, urban particulate matter) over 30 years. A search of PubMed (National Center for Biotechnology Information 2010) using the three terms “zinc,” “nanoparticle,” and “eosinophil” gave no hits, but there is one report of 25% eosinophils in the BALF of a human subject exposed accidentally to ZnO fume (Castet and Bouillard 1992).

In these experiments in the present study, we observed the eosinophilic responses when treatments were randomized across a group of rats, so it is unlikely to be explained by parasitic infections, especially because the rats were specific-pathogen free. In a separate experiment, we used ZnONP from another supplier, but these rats also showed eosinophilia in the BALF after 24 hr (data not shown). We found essentially the same extent of eosinophil influx when we used rat serum, human serum, or rat BALF as the dispersant, so we are confident that eosinophil recruitment is due to the ZnONP and not related to the method of dispersal. However, because this finding is so striking and unusual, it needs to be independently verified in other laboratories.

Our results with the four metal oxide NPs (CeO_2_NP, NiONP, ZnONP, and CuONP) suggest that each of these may have its own unique inflammatory footprint. The effects could arise from the NPs themselves, from soluble ions released from them, or from both. We previously showed that there can be differences in toxicity between different NPs of the same putative composition derived from different vendors (Lu et al. 2009). Such heterogeneity necessitates unique case-by-case hazard evaluation, risk assessment, and risk management. This would be especially true of CuONP and ZnONP, should our findings of an eosinophilic type response be borne out in exposed humans. Eosinophils play a key role in mediating asthma and other allergic conditions, and according to the World Health Organization (2010), 300 million individuals suffer from asthma globally. Some of these individuals are likely to be employed in workplaces where there is potential for NP exposure, which might result in recruitment of eosinophils to their lungs, greatly increasing their risk of asthma attacks.

There is a perceived need for rational toxicological (hazard) assessment of the large number of untested NPs and their variants (e.g., size, derivatization, composition), but there is also strong ethical and financial pressure to carry out such toxicological testing using *in vitro* approaches. Our findings sound a note of caution regarding this approach and strongly support the utility of *in vivo* models, because the variability in inflammatory responses and resulting pathology could never have been predicted or detected by *in vitro* assays. Although conventional *in vitro* assays might have found the panel of NPs to have a pattern of toxic potency similar to what we observed *in vivo*, such assays would never have revealed the underlying immunopathological mechanism, greatly enriching the hazard identification and hazard characterization of these materials (Clewell 2005). Other weaknesses of *in vitro* testing are well documented, including use of single cell lines to mimic complex tissue, lack of blood supply, and high doses (Donaldson et al. 2009).

## Figures and Tables

**Table 1:** Characterization of the eight NPs.

Nominal chemistry	CeO_2_NP	TiO_2_NP	CBNP^a^	SiO_2_NP	ZnONP^b^	CuONP^c^	NiONP	Beads^c^
Diameter (nm)	20–30	30–40	14	10	< 10	< 50	10–20	36
Surface area (m^2^/g)^d^	24.1	27.5	254	523.4	48.2	29	91.8	—
Mass (μg) per 150 cm^2^	625	545	59	25	310	515	163.5	94
Mass (μg) per 50 cm^2^	208	182	19.7	8.3	103	172	54.5	31.3
Hydrodynamic size (nm)^e^	88.1 ± 29.3	119.1 ± 39.6	78.0 ± 41.8	377.5 ± 170.2	306.3 ± 102.7	112.1 ± 17.3	92.5 ± 4.1	96.1 ± 11.8
Polydispersity^e^	0.148 ± 0.04	0.21 ± 0.07	0.18 ± 0.01	0.27 ± 0.05	0.27 ± 0.07	0.21 ± 0.03	0.21 ± 0.08	0.13 ± 0.02
Zeta potential (mV)^e^	−32.4 ± 5.6	−28.5 ± 5.2	−25.0 ± 5.0	−12.9 ± 3.7	−27.1 ± 1.4	−26.7 ± 12.5	−26.0 ± 5.0	40.3 ± 8.5
Endotoxin (pg/mL)^f^	ND	ND	ND	ND	ND	ND	ND	ND

ND, not detectable. Values are mean ± SD from four independent experiments. All chemicals were provided by Nanostructural and Amorphous Materials Inc. (Houston, TX, USA) except where noted.

a From Evonik Degussa GmbH (Frankfurt, Germany).

b From NanoScale Corporation (Manhattan, KS, USA).

c From Sigma-Aldrich Co. Ltd. (Gillingham, Dorset, UK).

d Determined by ParticlesCIC Ltd. except for CuO, which was supplied by Sigma-Aldrich Co. Ltd. (Gillingham, Dorset, UK).

e NPs were dispersed in PBS with 5% rat serum.

f Endotoxin detection limit < 10 pg/mL.

**Table 2:** Summary of inflammatory mediators, LDH, and total protein in BALF 24 hr (acute) and 4 weeks (chronic) after instillation of high-dose (150 cm^2^/rat) metal oxide NPs into rat lung.

BALF measure	VC	CeO_2_NP	NiONP	ZnONP	CuONP
Acute					

IL-1β (pg/mL)	0 ± 0	16.3 ± 7.5*	69.0 ± 25.5#	17.0 ± 7.7*	60.2 ± 12.5#
MIP-2 (pg/mL)	0 ± 0	226 ± 177*	643.5 ± 130.4#	11.1 ± 14.5	599.1 ± 264.0#
Eotaxin (pg/mL)	0 ± 0	11.3 ± 10.7	2.9 ± 2.5	714.4 ± 415.9#	54.4 ± 29.1#
IL-13 (pg/mL)	1.4 ± 0.3	3.0 ± 2.2	1.2 ± 0.6	44.2 ± 31.2#	0.6 ± 0.7
IFN-γ (pg/mL)	2.0 ± 4.0	0.9 ± 1.8	0 ± 0	0 ± 0	0 ± 0
LDH^a^	1.0 ± 0.01	3.3 ± 0.1#	3.5 ± 0.2#	12.3 ± 3.0#	6.4 ± 0.8#
Total protein (mg/mL)	0.19 ± 0.03	0.48 ± 0.13**	0.42 ± 0.08*	2.62 ± 0.63#	2.81 ± 1.14#
Inflammation (BALF and histology)	None	Neutrophilic/cytotoxic	Neutrophilic/cytotoxic	Eosinophilic/cytotoxic	Neutrophilic/eosinophilic/cytotoxic

Chronic					

IL-1β (pg/mL)	2.8 ± 1.4	8.4 ± 6.6	2.0 ± 2.3	0 ± 0	0 ± 0
MIP-2 (pg/mL)	8.2 ± 1.3	31.9 ± 10.2**	39.5 ± 9.0#	3.7 ± 3.3	7.3 ± 4.5
Eotaxin (pg/mL)	0 ± 0	0 ± 0	1.9 ± 1.4	0.2 ± 0.4	0 ± 0
IL-13 (pg/mL)	1.5 ± 1.2	1.2 ± 0.8	0.8 ± 0.9	1.8 ± 1.1	1.6 ± 0.4
IFN-γ (pg/mL)	0 ± 0	0.7 ± 1.3	34.8 ± 15.1#	11.4 ± 5.0**	0 ± 0
LDH^a^	1.0 ± 0.01	4.7 ± 2.4*	23.5 ± 1.9#	1.5 ± 0.6	1.3 ± 0.2
Total protein (mg/mL)	0.17 ± 0.07	0.42 ± 0.34	3.24 ± 0.39#	0.34 ± 0.01	0.37 ± 0.04
Inflammation (BALF and histology)	None	Neutrophilic/cytotoxic	Neutrophilic/lymphocytic/alveolar lipoproteinosis/cytotoxic	Eosinophilic/granulomatous/fibrosis	Granulomatous/fibrosis

VC, vehicle control. Data are mean ± SD (*n* = 4 per group). For full LDH and total protein levels, see Supplemental Material, Figures 1 and 2 (doi:10.1289/ehp.1002201); for the full cytokine profile, see Supplemental Material, Figures 5 and 6.

a Levels of LDH were expressed as fold changes compared with vehicle control.

* *p* < 0.05

** *p* < 0.01

# *p* < 0.001, compared with VC.
